# Calcium Supplement Use Is Associated With Less Bone Mineral Density Loss, But Does Not Lessen the Risk of Bone Fracture Across the Menopause Transition: Data From the Study of Women's Health Across the Nation

**DOI:** 10.1002/jbm4.10246

**Published:** 2019-11-15

**Authors:** Regan L Bailey, Peishan Zou, Taylor C Wallace, George P McCabe, Bruce A Craig, Shinyoung Jun, Jane A Cauley, Connie M Weaver

**Affiliations:** ^1^ Department of Nutrition Science Purdue University West Lafayette IN USA; ^2^ Department of Nutrition and Food Studies George Mason University Fairfax VA USA; ^3^ Think Healthy Group, Inc. Washington, DC USA; ^4^ Department of Statistics Purdue University West Lafayette IN USA; ^5^ Department of Epidemiology University of Pittsburgh Pittsburgh PA USA

**Keywords:** BONE DENSITY, CALCIUM, MENOPAUSE, OSTEOPOROSIS, WOMEN'S HEALTH

## Abstract

Diet is a modifiable factor that is related to bone mass and risk for fractures; however, the use of calcium supplements for bone health is controversial, with little scientific agreement. The purpose of this analysis was to estimate the change in lumbar spine and femoral neck BMD and the risk of bone fracture by the use of calcium supplements among the Study of Women's Health Across the Nation (SWAN) participants. SWAN is a multicenter, multiethnic, community‐based longitudinal cohort designed to examine the health of women across the menopause transition (*n* = 1490; aged 42 to 52 years at baseline in 1996 to 1997 and followed annually until 2006 to 2008). A mixed‐effect model for repeated measures was used to estimate annualized BMD change across time between supplement users and nonusers, unadjusted or fully adjusted (age, race, height, weight, menopausal status [pre‐, early peri‐, late peri‐, and postmenopausal], DXA scanner mode, alcohol intake, vitamin D supplement use, smoking, and physical activity) and a log‐linear model with repeated measures was used to estimate the relative risk of fracture by calcium supplement use. All models were also stratified by baseline menopausal status. In fully adjusted models, calcium supplement use was associated with less annualized loss of femoral neck BMD (−0.0032 versus −0.0040 g/cm^2^/year; *p* < .001) and lumbar spine BMD (−0.0046 versus −0.0053 g/cm^2^/year, *p* = 0.021) in the complete cohort. However, this protective association of calcium supplement use with BMD loss was significant only among premenopausal women (femoral neck: −0.0032 versus −0.0042 g/cm^2^/year; *p* = 0.002; lumbar spine: −0.0038 versus −0.0050 g/cm^2^/year, *p* = 0.001); no significant differences in BMD were observed among women who were early perimenopausal by calcium supplement use at baseline. No significant differences in the relative risk of fracture were observed, regardless of baseline menopausal status. The use of calcium supplements was associated with less BMD loss over more than a decade, but was not related to the risk of incident bone fracture across the menopause transition. © 2019 The Authors. *JBMR Plus* published by Wiley Periodicals, Inc. on behalf of American Society for Bone and Mineral Research.

## Introduction

Osteoporosis is described as a “progressive systemic skeletal disease characterized by low bone mass and microarchitectural deterioration of bone tissue, with a consequent increase in bone fragility and susceptibility to fracture.”^1^, ^2^ According to newly proposed criteria for diagnosis,^3^ the National Bone Health Alliance estimates that approximately 16% of men and 30% of women aged 50+ years in the United States have osteoporosis. An analysis by the National Osteoporosis Foundation (NOF) suggests that low bone mass and osteoporosis,^4^, ^5^ when combined, affects an estimated 56.6 million (54%) US adults aged 50+ years.^4^ The risk of osteoporosis and related fractures increases with age, especially among women after menopause.^6^, ^7^ Although many factors contribute to osteoporosis among postmenopausal women, the most significant cause is a decline in estrogen concentrations that leads to a rapid reduction in bone mass and structural deterioration of bone microarchitecture.

Given the rapid aging of our population,^8^ modifiable factors that contribute to bone health are of upmost importance. Several of the modifiable factors associated with age‐related bone disease include physical activity, smoking, and diet.^9^, ^10^, ^11^ Calcium is the dominant mineral present in bone and is considered a shortfall nutrient in the American diet per the 2015 to 2020 Dietary Guidelines for Americans.^12^ However, the relationship between calcium supplements with the risk of osteoporosis and related fractures has been controversial and various recommendations exist in the United States (Table 1). Data from the Women's Health Initiative (WHI) randomized controlled trial suggest that calcium in combination with vitamin D is associated with small, but significant improvement in BMD, but is not associated with a risk for bone fracture among postmenopausal women in intention‐to‐treat analysis.^13^ However, when women in the WHI were censored at the time of a protocol deviation, a significant risk reduction for hip fracture was observed (HR 0.71; 95% CI, 0.52 to 0.97).^13^ Thus, conflicting advice exists about whether to supplement with calcium for primary and secondary prevention of bone disorders associated with aging and loss of sex steroid hormones. Available data from clinical trials lack duration to adequately determine the effectiveness of calcium supplementation with regard to long‐term bone health across the menopause transition.^14^ Observational data from large longitudinal cohort studies in the United States generally do not capture measures of bone health directly and/or lack detailed information on a woman's menopausal status and transition.^14^ As a result, the US Preventive Services Task Force (USPSTF) found I‐level or “inconclusive” evidence to assess the benefits and harms of calcium and vitamin D supplementation, alone or combined, for the primary prevention of fractures in men and premenopausal women, and recommends against daily supplementation for the primary prevention of fractures in community‐dwelling postmenopausal women.^15^ Given the lack of clinical trial data in premenopausal women, the objective of this study was to estimate the annualized rate of BMD change and the risk of fractures with regard to the use of calcium supplements in a longitudinal cohort study specifically designed to assess bone health and the menopause transition.

**Table 1:** Current Guidelines and Position Statements for the Use of Calcium and/or Vitamin D for Bone Health

Institution	Nutrient	Outcome of interest	Guideline/position
National Osteoporosis Foundation (NOF; 2016)	Calcium	Peak bone mass development	“Strong” evidence for a beneficial effect, particularly during the late childhood and peripubertal years, a critical period for bone accretion
	Vitamin D	Peak bone mass development	“Moderate” evidence for a beneficial effect on peak bone mass accrual, but the generalizability is lacking
NOF (2015)	Calcium plus vitamin D	Risk of fracture	Supplementation reduced total and hip fractures by 15% and 30% in community‐dwelling and institutionalized postmenopausal women. The evidence supports the use of calcium plus vitamin D supplementation as an intervention for fracture risk reduction.
U.S. Preventive Services Task Force (USPSTF; 2013; 2018)	Calcium, vitamin D, and calcium plus vitamin D	Risk of fracture	The USPSTF recommends “against” daily supplementation with ≤400 IU of vitamin D and ≤1000 mg of calcium for primary prevention of fractures in community‐dwelling postmenopausal women. The USPSTF has no recommendations for postmenopausal women on doses >400 IU of vitamin D and >1000 mg of calcium for primary prevention of fractures in community‐dwelling postmenopausal women. The USPSTF has no recommendations for men and premenopausal women on calcium and vitamin D supplementation, alone or combined, for the primary prevention of fractures due to “inconclusive” evidence Supplementation of vitamin D alone or calcium plus vitamin D was not associated with reduced fracture incidence in community‐dwelling adults 50 years and older.
American Geriatrics Society (2014)	Vitamin D	Risk of fall and fracture	A serum 25(OH)D of 75 nmol/L should be a goal to achieve in older adults (65+ years). Clinicians are strongly advised to recommend at least 1000 IU of vitamin D per day plus calcium supplementation to community‐dwelling and institutionalized older adults to reduce risk of fractures and falls.

## Materials and Methods

This secondary data analysis utilized the Study of Women's Health Across the Nation (SWAN), a community‐based, multiclinical site (*n* = 7), longitudinal cohort of racially diverse women across the menopause transition. A detailed description of the SWAN design and methods has been previously published.^16^ Briefly, participants who were between the ages of 42 and 52 years at the onset of the study, were pre‐ or early perimenopausal, had an intact uterus and at least one ovary, and had not reported use of hormone therapy within the prior 3 months, were enrolled at baseline. The SWAN study protocol includes annual follow‐up visits to collect information on demographic, clinical, and anthropometric data. All participants provided written informed consent, and the entire SWAN protocol was approved by all SWAN site institutional review boards.

**Figure 1:**
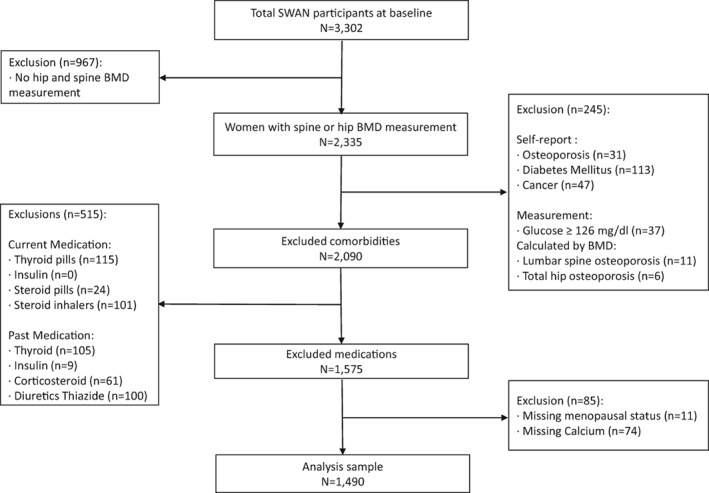
Flowchart of the analysis sample at baseline. Participants are from the Study of Women's Health Across the Nation (SWAN).

The SWAN bone substudy was conducted within five of the seven clinical sites to assess skeletal changes during the menopausal transitions across different races/ethnicities. Data in this analysis came from the publicly available repository (ie, baseline through visit 10) and includes participants who had complete data on DXA measurements of the femoral neck and/or lumbar spine at baseline (*n* = 2335). Women who had chronic disease associated with bone alterations at baseline were excluded, including osteoporosis (*n* = 31 from self‐report^17^; and *n* = 17 because of BMD *T*‐scores less than 2.5 standard deviations from the referent population^18^, ^19^), diabetes (*n* = 113 from self‐report; and *n* = 37 based on a fasting glucose level ≥126 mg/dL),^20^ and cancer (*n* = 47 from self‐report).^21^ Women who had a recent or past history of medication use associated with alterations in bone, specifically: thyroid medications (*n* = 225),^22^ steroids and corticosteroids (*n* = 286),^23^ thiazide diuretics (*n* = 101, not collected as current only past history available),^24^ and insulin (*n* = 9),^20^ were excluded. Lastly, women with missing data on baseline menopausal status and dietary supplement use were also excluded (*n* = 74). Therefore, our final analytic sample at baseline included 1490 women (376 African American, 170 Chinese, 197 Japanese, 747 Caucasian) at baseline (Fig. 1).

Demographic data (ie, age, race/ethnicity) were obtained at baseline via standardized questionnaires. Race/ethnicity categorization as provided in the dataset and self‐reported was Black/African American, Chinese/Chinese American, Japanese/Japanese American, and Caucasian/White non‐Hispanic; no Hispanics participated in the SWAN bone substudy.

Height (in centimeters) and weight (in kilograms) were measured annually from baseline through visit 10 via standardized protocols across all sites. Both height and weight measurements were obtained without shoes and with light indoor clothing, using a stadiometer (for height) and a calibrated scale (for weight), and were used to calculate BMI (g/cm^2^).^25^ BMD measurements at the lumbar spine and femoral neck were obtained annually using DXA via Hologic instruments (Hologic Inc., Waltham, MA, USA); the Pittsburgh and Oakland sites used the Hologic 2000 model and the remaining three sites used the Hologic 4500A densitometer.^26^ The recommended machine calibration correction factors were applied. Osteodyne (Research Triangle Park, NC, USA) positioning devices were used to improve the reproducibility of hip measurements.^27^ The SWAN DXA quality‐control procedures have been published in extensive detail elsewhere.^28^ Bone fracture data were obtained by self‐report using the interview questionnaire from visit 1 to visit 10. The fractures not typically associated with osteoporosis (eg, face, skull, fingers, and toes) were excluded from the fracture analysis.^29^, ^30^ Fracture history since the age of 20 years was collected from the baseline interview questionnaires.

Self‐reported menopausal status was collected at each visit; sex steroid hormones were also evaluated relative to the self‐report with excellent agreement (data not shown). Menopausal status was categorized as premenopause (bleeding in the past 3 months with the same pattern since last year), early perimenopause (bleeding in the past 3 months with decreased menstrual regularity in the past year), late perimenopause (no bleeding for 3 to 11 months), and postmenopausal (no bleeding in the past 12 months) based on the annual reports about menstrual bleeding patterns. A variable was constructed to assess baseline smoking status from a self‐administered questionnaire: current smoking status (individuals who reported >20 packs of cigarettes in their lifetime and currently smoked), former smoking status (individuals who reported >20 packs of cigarettes in their lifetime, but did not currently smoke), or never smoking status (individuals who reported have not smoked a total of at least 20 packs of cigarettes over their lifetime). Physical activity was assessed in comparison with peers and was categorized as much less, somewhat less, the same, somewhat more, and much more.

Information on current use of dietary supplements containing calcium and vitamin D and consumption of alcohol was obtained as part of a standardized annual follow‐up interview questionnaire except at baseline, when this information was only collected as part of a food frequency questionnaire (FFQ).^24^ SWAN collected the modified Block FFQ, which was used only at baseline to define calcium supplement users. At baseline, self‐reported calcium‐containing supplement use was dichotomized into nonuser (do not take any from the interview questionnaire data or less than 300 mg from the FFQ data) and user (≥1 day per week from the interview questionnaire data or more than 300 mg from the FFQ data). The 300‐mg criterion was selected because it represents what is likely achieved from a serving of milk or dairy product, not a multivitamin mineral supplement that contains a typical dose of approximately 130 mg of calcium. At time points other than baseline and visit 5, self‐reported calcium and vitamin D supplement use was categorized into nonuser and user based on the standardized questionnaire; visit 5 supplement use was imputed using data from visit 4 and/or visit 6 (if the data were missing at visit 4).

Self‐reported alcohol consumption was also categorized into nonconsumer (did not drink any beer, wine, liquor, or mixed drinks from the interview questionnaire or average daily servings =0 from FFQ data) or consumer (self‐reported drink in interview questionnaire or average daily servings >0 from FFQ data).

### Statistical analysis

All statistical analyses were conducted using SAS 9.4 (SAS Institute, Inc., Cary, NC, USA). Differences in subject characteristics for baseline calcium supplement groups were analyzed using *t* tests with Satterthwaite approximation for the *p* value for continuous variables, and chi‐square tests for categorical variables.

The annualized rate of loss in BMD of the femoral neck and lumbar spine by time‐varying calcium dietary supplement use was estimated using linear mixed models. SAS PROC MIXED for repeated measures was used with KENWARDROGER adjustment to compute denominator degrees of freedom and a compound symmetry covariance structure. The length of time between BMD scan date and baseline scan date was used as the time variable in the model. The interaction term between time and calcium supplement use estimates the difference in annualized BMD loss between users and nonusers.

Set‐wise methods were used to build three mixed models: (1) unadjusted; (2) adjusted for a priori biological factors (model 1: race [fixed], baseline height [fixed], weight [time‐varying], menopausal status [time‐varying], baseline age [fixed], and DXA scanner mode [time‐varying]); and (3) adjusted for additional lifestyle factors (model 2: baseline smoking status [fixed], baseline physical activity [fixed], alcohol consumption [time‐varying)], and vitamin D use [time‐varying]).

For fracture analysis, we used a Poisson regression analysis in SAS PROC GENMOD with a log‐link function to calculate the relative risk (RR) of incident fractures and 95% confidence intervals (CIs) for calcium dietary supplement users and nonusers. The fully adjusted model controlled for the same covariates with model 2 above, except for excluding scanner mode and baseline age and including time‐varying age and fracture history.

Participants were stratified into premenopause and early perimenopause based on their menopausal status at baseline to examine a potential differential relationship between menopausal status and calcium supplement usage with regards to BMD loss and fracture risk. Fracture models were also examined in Caucasian women only. The same statistical models described above for BMD and fracture risk were applied to the stratified analysis. Statistical significance was set at *p* value <0.01 to account for multiple comparisons.

### Censoring

Women who became pregnant or postmenopausal as a result of bilateral salpingo‐oophorectomy, self‐reported diabetes, or who had a fasting glucose level ≥126 mg/dL, self‐reported cancer, or self‐reported over‐/underactive thyroid since the last visit were censored from the time of report until the end of the study. Participants who were still in their pre‐ or early perimenopausal status from visit 5 onwards (*n* = 536) were censored from the BMD loss rate analysis, but not from the fracture analysis.

## Results

At baseline, 19% of the sample reported use of calcium supplements, but no differences in mean calcium intake from food sources between users and nonusers of calcium supplements were observed. At subsequent visits, the percentage of users ranged from 22% to 39% (Supplemental Table S1). At baseline, calcium users were more likely to be older, use vitamin D supplements, have a lower BMI and BMD, and be Caucasian and of Japanese descent (Table 2). Calcium supplement users were less likely to be current smokers and were more likely to report a higher frequency of physical activity compared with nonusers; however, no differences were observed for menopausal status or the use of alcohol. Twenty‐two percent of calcium supplement users reported a history of prior bone fracture, whereas 16% of nonusers reported prior bone fracture, but the difference did not reach statistical significance (*p* = 0.04).

**Table 2:** Baseline Characteristics of Calcium Supplement Users and Nonusers^a^

	Users (*n* = 283)	Nonusers (*n* = 1207)	*p* value
Age (year), mean (SD)	46.3 (2.8)	45.7 (2.6)	<.001
Height (cm), mean (SD)	162.9 (6.6)	162.6 (6.7)	0.411
Weight (kg), mean (SD)	67.8 (16.4)	71.1 (18.0)	0.005
BMI (kg/cm^2^), mean (SD)	25.5 (5.6)	26.9 (6.2)	<.001
Dietary calcium (mg), mean (SD)	750 (427)	751 (419)	0.959
Vitamin D supplement use, *n* (%)			<.0001
User	23 (2.1)	27 (10.2)	
Nonuser	1061 (97.9)	238 (89.8)	
BMD (g/cm^2^), mean (SD)			
Femoral neck	0.92 (0.1)	0.96 (0.1)	<.001
Lumbar spine	1.05 (0.1)	1.07 (0.1)	0.003
Race, *n* (%)			<.001
African‐ American	32 (11.3)	344 (28.5)	
Chinese	25 (8.8)	145 (12.0)	
Japanese	53 (18.7)	144 (11.9)	
Caucasian	173 (61.1)	574 (47.6)	
Menopausal status, *n* (%)			0.308
Premenopause	154 (54.4)	697 (57.8)	
Early perimenopause	129 (45.6)	510 (42.3)	
Smoking status, *n* (%)			0.006
Current smoker	26 (9.2)	199 (16.6)	
Former smoker	80 (28.4)	288 (24.0)	
Nonsmoker	176 (62.4)	713 (59.4)	
Physical activity, *n* (%)			0.208
Much less	26 (9.3)	160 (13.3)	
Somewhat less	69 (24.6)	327 (27.2)	
The same	88 (31.3)	341 (28.3)	
Somewhat more	81 (28.8)	297 (24.7)	
Much more	17 (6.0)	79 (6.6)	
Alcohol, *n* (%)			0.300
Alcohol consumer	152 (53.7)	607 (50.3)	
Nonconsumer	131 (46.3)	600 (49.7)	
Fracture history			0.038
Had a broken bone	61 (21.7)	198 (16.5)	
Never broken a bone	220 (78.3)	1003 (83.5)	

a Baseline missing data: height (*n* = 13), weight (*n* = 2), BMI (*n* = 14), smoking status (*n* = 8), spine BMD (*n* = 13), hip BMD (*n* = 6), physical activity (*n* = 5), fracture history (*n* = 8), vitamin D supplement use (*n* = 141).

Although users of calcium supplements at baseline had a lower femoral neck and spine BMD, they had a lower rate of loss of BMD across time, after adjustment for potential confounding variables (Table 3). When stratified by baseline menopausal status, the protective association of calcium use and BMD loss was significant among premenopausal women for both femoral neck and lumbar spine (*p* = 0.002 and 0.001, respectively), but not among early perimenopausal women (*p* = 0.086 and 0.875, respectively).

**Table 3:** Annualized Rate of BMD Loss by Calcium Supplement Use From Baseline to Visit 10

	Annualized rate of BMD loss (g/cm^b^/year)					
	Femoral neck			Lumbar spine		
	User	Nonuser	*p* value	User	Nonuser	*p* value
Complete cohort						
Unadjusted	−0.00481	−0.00502	0.336	−0.01038	−0.01021	0.573
Model 1^a^	−0.00312	−0.00395	<.001	−0.00455	−0.00527	0.010
Model 2^b^	−0.00321	−0.00400	<.001	−0.00463	−0.00529	0.021
Premenopause (at baseline)						
Unadjusted	−0.00488	−0.00495	0.825	−0.01002	−0.01001	0.988
Model 1^a^	−0.00315	−0.00413	0.002	−0.00373	−0.00495	<.001
Model 2^b^	−0.00324	−0.00422	0.002	−0.00380	−0.00501	0.001
Perimenopause (at baseline)						
Unadjusted	−0.00473	−0.00510	0.251	−0.01073	−0.01367	0.502
Model 1^a^	−0.00337	−0.00404	0.052	−0.00550	−0.00569	0.652
Model 2^b^	−0.00345	−0.00405	0.086	−0.00559	−0.00566	0.875

a Model 1 adjusted for race, baseline age, baseline height, time‐varying weight, time‐varying menopausal status, and time‐varying scanner mode.

b Model 2 adjusted for all covariates included in model 1 as well as baseline physical activity, baseline smoking status, time‐varying alcohol consumption, and time‐varying vitamin D supplement use.

Over approximately 10 years of follow‐up, 116 women experienced 140 fractures. No differences in the RR of bone fractures were observed by time‐varying calcium supplement use, regardless of the model applied (Table 4). Furthermore, results did not vary when examined separately by menopausal status. Lastly, no differences in the RR of fractures were observed between users and nonusers of calcium supplements when Caucasian and non‐Caucasian women were examined separately.

**Table 4:** Adjusted Relative Risk (RR) and 95% Confidence Intervals (CIs) for Incident Fractures by Calcium Supplement Use From Baseline to Visit 10

User versus nonuser	RR (95% CI)	*p* value
Complete cohort		
Unadjusted	1.17 (0.80–1.71)	0.426
Fully adjusted^a^	1.16 (0.76–1.77)	0.498
Premenopause (at baseline)		
Unadjusted	1.07 (0.65–1.75)	0.797
Fully adjusted^a^	1.18 (0.67–2.06)	0.570
Early perimenopause (at baseline)		
Unadjusted	1.31 (0.73–2.36)	0.364
Fully adjusted^a^	1.12 (0.58–2.16)	0.733
Caucasian		
Unadjusted	1.33 (0.82–2.14)	0.248
Fully adjusted^a^	1.30 (0.77–2.17)	0.325
Non‐Caucasian		
Unadjusted	0.97 (0.52–1.82)	0.926
Fully adjusted^a^	1.03 (0.48–2.18)	0.943

a Adjusted for race, time‐varying age, time‐varying weight, baseline height, time‐varying menopausal status, baseline smoking status, time‐varying alcohol use, baseline physical activity, fracture history, and time‐varying vitamin D supplement use.

## Discussion

The menopausal transition represents a dynamic time in which bone mass is known to deteriorate rapidly. Little data are available to understand if any primary nutritional prevention strategies can mitigate the risk of bone loss longitudinally across the menopausal transition. The SWAN data indicate that although calcium supplement users had lower femoral neck and lumber spine BMD at baseline, they had a lower annualized rate of femoral neck and lumber spine BMD loss over time after adjusting for potential confounding variables when compared with nonusers. When examined separately, the results were consistent among women who were in premenopause at baseline, but not among women in early perimenopause at baseline. Calcium users lost BMD at a rate that was 20% and 12% less than that of nonusers at the femoral neck and lumbar spine, respectively. However, the RR of incident fractures was not different between calcium supplement users and nonusers, and these results were consistently null when stratified by menopausal status and when limited to Caucasian women, the majority of whom were represented in the fracture group. This could be an artifact of the small number of fractures observed in our analytical sample (*n* = 140), but may also represent an interesting finding. Calcium supplement users at baseline were more likely to have experienced a prior fracture(s), have lower BMI and BMD, and be of Caucasian descent: All of which are established risk factors for fracture, suggesting that this group may have been taking calcium supplements because they were aware of their higher risk of bone fracture. Thus, one speculative explanation of the null finding for fracture risk is that it may represent a success of calcium supplement users who were at higher risk of bone fracture at baseline because they did not significantly differ from nonusers who were at lower risk for bone fracture at baseline. Clinical trial data in pre‐ and perimenopausal are needed, however, to confirm such potential explanations.

Nutrition is an essential component in health promotion and disease prevention and management, but is not considered a curative therapy for osteoporosis per se. In an era that requires the practice of evidence‐based medicine, clinical recommendations are often based on systematic reviews and meta‐analyses. However, the reviews contain data that provide only a snapshot in time (eg, postmenopausal state) as to the potential effects of interventions such as calcium supplementation on bone health. Randomized controlled trials (RCTs) are considered the gold standard from a clinical research paradigm; nevertheless, there is a shortage of high‐quality diet‐related intervention trials utilizing BMD and fractures as primary outcomes, thereby forcing the use of observational research to inform research and clinical practice. The largest RCT to date was from the WHI, which focused on postmenopausal women (*n* = 36,282); it found that calcium (1 g/d) and vitamin D (400 IU/d) supplements for an average intervention of 7 years did not significantly reduce fracture incidence of hip or total fractures compared with the placebo group.^13^ However, women were allowed to consume their own supplements. When the data were reanalyzed excluding those women taking their own supplements and those less than 85% compliant with the intervention, calcium and vitamin D supplementation significantly reduced hip fracture.^31^ When this analysis was included in a meta‐analysis, calcium and vitamin D supplementation was found to reduce hip fracture by 30%.^32^ Thus, many conflicting opinions exist about whether to recommend calcium supplements for bone health. Furthermore, data examining critical time points at which calcium supplements may be most useful to mitigate bone loss are scarce. A recent systematic review of the role of individual nutrients, food patterns, special issues, contraceptives, and physical activity by the NOF found A‐level or “strong” evidence for a beneficial effect of calcium intake on peak bone mass attainment, particularly during the late childhood and peripubertal years—a critical period for bone accretion.^9^ A separate systematic review by the NOF concluded that calcium with vitamin D supplementation reduced total fractures and hip fractures by 15% and 30%, respectively, in community‐dwelling postmenopausal women who were at least 80% adherent to the supplement regimen.^32^

The USPSTF found I‐level or “inconclusive” evidence to assess the benefits and harms of calcium and vitamin D supplementation, alone or combined for the primary prevention of fractures in men and premenopausal women and for doses greater than 400 IU of vitamin D and 1000 mg of calcium for the primary prevention of fractures in community‐dwelling postmenopausal women. The USPSTF currently recommends against daily supplementation with 400 IU or less of vitamin D and 1000 mg or less of calcium for primary prevention of fractures in community‐dwelling postmenopausal women.^15^ The American Geriatrics Society advises clinicians to recommend at least 1000 IU of vitamin D per day and calcium supplementation to community‐dwelling adults age 65+ years to reduce the risk of fractures and falls when micronutrient requirements are not being met through diet alone.^33^

### Strengths and limitations

The primary strength of this analysis is its incorporation of information on calcium supplement use, many confounding variables, BMD, and fractures across the menopause transition. Therefore, we could highlight the early role of calcium supplement use on bone health and examine changes in BMD over a decade, controlling for multiple confounders.^14^ However, there are many limitations and caveats to consider when interpreting these data. First, this analysis was limited to the public‐use data set that only extended to visit 10 and did not include information on the study site. Second, fractures were self‐reported from baseline to visit 5. Nonetheless, fractures after visit 6 were confirmed by a medical records review; previous comparison of the self‐report and medical record‐adjudicated fracture determined that self‐report yielded a false–positive finding of <5%.^34^ Because of small sample sizes, classification of traumatic fractures was not differentiated. Third, dietary supplement use was self‐reported; currently, no methods exist to validate self‐reported calcium or vitamin D supplement use, such as recovery biomarkers.^35^ Fourth, although we adjusted for many possible confounders, residual confounding and missing data are always a limitation in observational research. Although no differences in baseline calcium from foods and beverages were observed, dietary calcium intake was not available across all study years. Finally, as these data are limited to a cohort that only included non‐Hispanic women, our findings may not be applicable to Hispanic women or men.

## Conclusions

In the absence of clinical trial data, observational research is crucial for examining potential relationships between nutrition and bone across the menopausal transition. Given the limitations described above, this work cannot be used to demonstrate a cause‐and‐effect relationship^36^; but, with careful consideration of the causal criteria in nutritional epidemiology, it can help address research questions for which RCT data are lacking or questions that RCTs are not suitable to address.^37^ Longitudinal cohort studies like this analysis are important to study changes in bone trajectory over time, and are critically needed to tailor dietary recommendations based on age and life‐stage.^14^ The SWAN data suggest that though the use of calcium dietary supplements was associated with a slower rate of decline in femoral neck and lumber spine BMD among middle‐aged and older women, especially among those in premenopause, there was no association between calcium dietary supplements and incidence of bone fracture.

## Disclosures

RLB has received funding from the NIH/NCI, and serves as a scientific consultant to the NIH, Office of Dietary Supplements. She has received travel support from the Council of Responsible Nutrition, the American Society of Nutrition (as well as an honorarium), and the New York Academy of Sciences to present her research on nutrient intakes and supplementation. She has served as a consultant to Columbia University and RTI International on the 2016 Feeding Infants and Toddlers Study data (funded by Nestle R&D). TCW has received research support from Pfizer Consumer Healthcare and scientific consulting fees from several food companies. All of his conflicts are listed at http://www.drtaylorwallace.com. CMW is on the scientific advisory boards of yogurt in nutrition (YINI) and FDA, and the Board of Trustees of ILSI. PZ, SJ, GM, and JAC have no conflicts of interest to disclose.

## Supporting information


**Table S1** Calcium Supplement Use From Baseline to Visit 10 for the Population in This Analysis
