# Supplementary material for: Calcium Supplement Use Is Associated With Less Bone Mineral Density Loss, But Does Not Lessen the Risk of Bone Fracture Across the Menopause Transition: Data From the Study of Women's Health Across the Nation
Source: JBMR Plus. 2019 Nov 15;4(1):e10246. doi: 10.1002/jbm4.10246 (PMC6957983; doi:10.1002/jbm4.10246)
Supplement: Supplementary file 1 — Table S1 Calcium Supplement Use From Baseline to Visit 10 for the Population in This Analysis [file JBM4-4-e10246-s001.docx]

**Supplemental Table 1**: Calcium supplement use from baseline to visit 10 for the population in this analysis.

|  | Baseline^a^ | Visit 01 | Visit 02 | Visit 03 | Visit 04 | Visit 05^b^ | Visit 06 | Visit 07 | Visit 08 | Visit 09 | Visit 10 |
| --- | --- | --- | --- | --- | --- | --- | --- | --- | --- | --- | --- |
| *n* | 1490 | 1310 | 1241 | 1158 | 1116 | 593 | 582 | 516 | 307 | 478 | 487 |
| Calcium user | 283  (19.0%) | 349 (26.6%) | 382 (30.8%) | 413 (35.7%) | 409 (36.7%) | 218 (36.8%) | 226 (38.8%) | 179 (34.7%) | 107 (34.9%) | 149 (31.2%) | 105 (21.6%) |
| Vitamin D User | 50  (3.7%) | 50 (3.8%) | 58 (4.7%) | 75 (6.5%) | 90 (8.1%) | 47  (8.1%) | 41 (7.0%) | 34 (6.6%) | 22 (7.2%) | 47 (9.8%) | 46 (9.5%) |

^a^ Calcium supplement use information was from food frequency questionnaire (FFQ); Vitamin D supplement use information was imputed using data from Visit 01.

^b^ Supplement use information was imputed using data from Visit 04 and/or Visit 06.
